# Rumen microbiota helps Tibetan sheep obtain energy more efficiently to survive in the extreme environment of the Qinghai–Tibet Plateau

**DOI:** 10.3389/fmicb.2024.1431063

**Published:** 2024-07-24

**Authors:** Xiukun Wu, Gaosen Zhang, Wei Zhang, Jianwei Zhou, Haitao Cong, Guo Yang, Guangxiu Liu

**Affiliations:** ^1^Key Laboratory of Ecological Safety and Sustainable Development in Arid Lands, Northwest Institute of Eco-Environment and Resources, Chinese Academy of Sciences, Lanzhou, China; ^2^Key Laboratory of Extreme Environmental Microbial Resources and Engineering, Lanzhou, China; ^3^State Key Laboratory of Herbage Improvement and Grassland Agro-ecosystems, College of Pastoral Agriculture Science and Technology, Lanzhou University, Lanzhou, China; ^4^Shandong Huakun Rural Revitalization Institute Co., Ltd., Jinan, China

**Keywords:** Tibetan sheep, small-tailed Han sheep, rumen bacterial, low-protein diet, energy levels

## Abstract

**Introduction:**

T-sheep and H-sheep exhibit different environmental adaptability and production performance. The rumen microbiome has co-evolved with hosts and plays a vital role in nutrient digestion and energy metabolism. In our previous study, we found that T-sheep have a higher efficiency in energy metabolism than H-sheep, but the rumen microbial community remains unclear.

**Methods:**

In this study, we determined the rumen bacterial profile and rumen fermentation parameters to reveal the bacterial profiles and predictive functions among breeds and diets with four different energy levels, as well as the correlation between bacterial profiles and rumen fermentation characteristics.

**Results:**

The results showed that the rumen total volatile fatty acids (VFAs), acetate, butyrate, total branched-chain VFAs, iso-butyrate, and iso-valerate were higher in T-sheep than H-sheep. The alpha diversity of ruminal bacteria is not affected by dietary energy, but it shows a distinction between the sheep breeds. Specifically, T-sheep rumen bacteria exhibit higher alpha diversity than H-sheep. The beta diversity of ruminal bacteria is not influenced by dietary energy or sheep breeds, indicating similar communities of ruminal bacteria between different diets and sheep breeds. The phyla of Bacteroidetes and Firmicutes predominate in the rumen, with a higher relative abundance of Firmicutes observed in T-sheep than H-sheep. The two most abundant genera in the rumen were *Prevotella* 1 and *Rikenellaceae* RC9 gut group. *Prevotella* 1 is the predominant bacterial genus in the rumen of H-sheep, while the *Rikenellaceae* RC9 gut group dominates in the rumen of T-sheep. Microbial co-occurrence network analysis reveals that variations in rumen fermentation characteristics result from differences in module abundance, with a higher abundance of VFA-producing modules observed in the rumen of T-sheep. Microbial function prediction analysis showed that dietary energy rarely alters the functional composition of rumen bacteria. However, there were differences in the functions of rumen bacteria between sheep breeds, with T-sheep showing a greater emphasis on energy metabolism-related functions, while H-sheep showed a greater emphasis on protein metabolism-related functions.

**Discussion:**

These findings provide evidence of the special rumen microbial community that helps T-sheep efficiently obtain energy from low-protein and low-energy diets, enabling them to survive in the extreme environment of the Qinghai–Tibet Plateau.

## 1 Introduction

The rumen is a complex and dynamic ecosystem harboring a diverse collection of microorganisms, including bacteria, archaea, fungi, and protozoa. Among these microorganisms, bacteria constitute the largest proportion of the ruminal microbiome ecosystem (Puniya et al., 2015). Rumen microorganisms play a pivotal role in the nutritional metabolism of ruminants, helping the host to digest plant ingredients into volatile fatty acids and synthetic microbial protein that can be absorbed by the host (McCann et al., 2014). In addition, they also impact ruminant production performance and health traits (Rey et al., 2014). To enhance feed conversion efficiency, improve animal productivity and wellbeing, and even reduce methane emissions, it is necessary to obtain a deep understanding of the dynamics and function of rumen microorganisms (Naas and Pope, 2019). To date, numerous studies have found that rumen microorganisms are dramatically influenced by many aspects, such as diet composition (Mohammed et al., 2014; Henderson et al., 2015; Liu et al., 2019), growth stages (Jami et al., 2013), breeds and species (Weimer et al., 2010; Weimer, 2015), host genetics (Li F. et al., 2019), and living environment (Henderson et al., 2015). Notably, the dietary patterns, host genetics, and living environment are diverse around the world. Further study on rumen microbiome profile and function between the different ruminants treated with the same or different dietary patterns while living in various environments, especially in extreme environments, is needed. The intestinal microorganisms, including rumen microorganisms, of plateau animals play a crucial role in adapting to high-altitude environments. The gut microbiome of plateau animals exhibits high diversity and cellulolytic activity, which supports forage digestion and the production of short-chain fatty acids essential for maintaining energy levels in the face of nutritional deficiencies, enabling plateau animals to survive on sparse and low-quality forage (Li et al., 2022; Liu et al., 2022a,b).

The Qinghai–Tibet Plateau (QTP) is the largest plateau in China and the highest in the world. It is called the “Roof of the World” and the “Third Pole.” Extreme environmental conditions such as high altitude, low oxygen partial pressure, low temperature, and high ultraviolet radiation in the QTP challenge the survival of humans and mammalian species (Royden et al., 2008). Tibetan sheep (T-sheep) (*Ovis aries*) is an important species in addition to yak on the QTP, numbering 50 million head, and an important source of meal, milk, and leather for nomadic pastoralists on the QTP. T-sheep graze on the rangeland all year round, without receiving supplements. With living in this harsh environment and poor forage available in the cold season, the reproductive performance of T-sheep is relatively low (a single lamb per year) (Zhou J. et al., 2019). Small-tailed Han sheep (H-sheep) (*Ovis aries*) is a fine variety breed raised in feedlots in northern China, numbering 5.53 million. They were introduced to the plateau in the 1980s because of their characteristics of fast growth, high resistance to rough feeding, high reproductive performance (producing 2–5 lambs per lambing and 2 lambing per year), high slaughter rate, good meat, and leather quality (Wang et al., 2017). H-sheep are mainly maintained indoors at lower altitudes than Tibetan sheep and only graze natural pasture in summer (Zhou J. et al., 2019). Due to the different living environments, T-sheep and H-sheep have different physiological characteristics. T-sheep are often exposed to low-energy and low-protein diets, with husbandry for thousands of years, which may suggest they have evolved highly efficient in energy metabolism. H-sheep highly productive performance requires more protein, which may suggest they are able to metabolize large amounts of protein.

Our previous study tested the T-sheep and H-sheep metabolic characteristics treated with low-protein and four different low-energy level diets and found that the average daily gain (ADG) was negative in the H-sheep at the 6.73 and 7.65 MJ/kg DM diets, but it was only negative in the T-sheep at the 6.73 MJ/kg DM diet. T-sheep had a greater ADG than H-sheep at the 7.65, 8.57, and 9.49 MJ/kg DM diets and lost less body mass on the 6.73 MJ/kg DM diet. In addition to the difference in average daily gain, other indicators such as digestibility of dry matter, organic matter, gross energy, neutral and acid detergent fibers, microbial nitrogen production, and volatile fatty acids (VFAs) were greater in T-sheep than H-sheep. This suggests that T-sheep were better able to cope with low-protein, low-energy diets than H-sheep (Zhou J. et al., 2019). These facts indicated that T-sheep are more efficient in energy metabolism than H-sheep. Rumen microbiome has co-evolved with hosts (Zhang et al., 2016) and plays a vital role in nutrient digestion and energy metabolism, but in our previous study, the ruminal microbial community structure remains unclear.

In this study, we used the 16S rRNA gene high-throughput sequencing technology to investigate the ruminal bacterial community structure and function variation by treating with low-protein and four different energy level diets between two sheep breeds. We aim to answer the three questions as follows: (1) How do ruminal bacteria change between diets with four different energy levels and among the different breeds? (2) What are the differences in ruminal bacterial functions and metabolic pathways among different breeds and energy levels of diets? (3) Does the T-sheep rumen bacterial function emphasize energy metabolism more for their adaptation to living in cold areas, while the H-sheep rumen bacterial function emphasizes protein metabolism more for faster growth and higher reproductive capacity? Those answers would help us to understand energy metabolism and nitrogen utilization between the H-sheep and T-sheep from the rumen bacterial aspect, so as to provide a scientific basis for improving the performance of T-sheep and the environmental adaptability of H-sheep.

## 2 Materials and methods

### 2.1 Study sites, animals, and diets

This experiment was carried out at the Wushaoling Yak Research Facility of Lanzhou University (37.21°N 102.86°E, altitude 3,154 m) which was located in northeastern Qinghai–Tibetan Plateau from October 2016 to January 2017. During the trial, the average air temperature was 6°C, and the average relative humidity was 76%. Five Tibetan wethers (47.7 ± 2.46 kg BW) of Oula type and five small-tailed Han wethers (46.2 ± 3.42 kg BW) were used in this study, which were purchased from a nearby Tibetan herder and a feedlot, respectively. The sheep were provided with fresh water *ad libitum* and kept individually in metabolic cages within a roofed shelter. Four energy level diets were fed according to a 4 × 4 Latin square, with one energy level repeated in each period (Sarraseca et al., 1998). A 20-day adaptation period allowed the animals to familiarize themselves with the experimental procedures before the formal study. The formal study included 4 periods, each period lasted 20 days.

The sheep were fed in two equal portions at 08:00 and 18:00, with a total of 818 g dry matter (DM) of pellets daily. This feed intake is 80% of the free feed intake. The four energy levels of diet with metabolizable energy (ME) were 6.73, 7.65, 8.57, and 9.49 MJ/kg DM, respectively. The ingredient and chemical composition of the experimental diets are the same as in the previous study (Zhou J. W. et al., 2019). The crude protein (CP) content of diets is approximately 7%. This CP level is similar to the average CP content in forages during the cold season of the Qinghai–Tibetan Plateau (Zhou J. et al., 2019). According to NRC (2007), the 6.73 and 7.65 MJ/kg DM diets provided energy were lower than sheep maintenance body mass requirements.

### 2.2 Rumen fluid collection and chemical analysis

At the end of each study period (day 20, day 40, day 60, and day 80), approximately 50 mL of rumen fluid was collected before the morning feed by using an oral stomach tube from each sheep. Each rumen fluid was divided into two parts: one part was transferred into a sterilized container and stored at −80°C for microbial community analysis, and the other part was strained through four layers of cheesecloth for pH and volatile fatty acids (VFA) measurement. The rumen fluid pH was measured by using a pH meter (PB-10, Sartorius Co., Germany), and VFA was analyzed using gas chromatography (SP-3420A, Beifenrili Analyzer 134 Associates, Beijing, China) with a capillary column (AT-FFAP: 30 m × 0.32 mm × 0.5 μm). The column oven of the gas chromatography was initially set at a temperature of 90°C, which was then ramped up to 120°C at a rate of 10°C per minute and maintained for a duration of 3 min. Subsequently, the temperature was further increased to 180°C at the same rate and held steady for an additional period of 5 min.

### 2.3 DNA extraction, PCR amplification, and high-throughput sequencing

The genomic DNA of the rumen fluid was extracted using the OMEGA E.Z.N.A stool DNA kit according to the manufacturer’s instructions. The 16S rRNA gene V3–V4 variable regions were selected for the characterization of bacterial diversity, using primers 341F (5′-CCTAYGGGRBGCASCAG-3′) and 806R (5′-GGACTA CNNGGGTATCTAAT-3′) to amplified target gene. The PCR reaction was amplified by using TransStart Fastpfu DNA polymerase (TrasnsGen, Beijing, China) on the GeneAmp 9700 instrument (ABI, CA, USA). The presence and sizes of the PCR amplification products were determined by agarose (2.0%) gel electrophoresis. Amplicons were purified using the AxyPrep DNA Gel Extraction Kit (Axygen, CA, USA) as directed by the manufacturer’s instructions. The concentrations of amplicons were quantified by QuantiFluorTM-ST (Promega, CA, USA), and equal amounts of all amplicons were mixed in a single tube, processed with the TruSeq RNA and DNA Sample Preparation Kit (Illumina, CA, USA), and then paired-end sequenced on an Illumina HiSeq platform.

### 2.4 Sequencing data processing

All raw read sequences were paired-end assembled using Trimmomatic and FLASH (Salzberg and Magoč, 2011). Reads that could not be assembled were discarded. The chimeric and singleton sequences were identified and removed, and operational taxonomic units (OTUs) were clustered with 97% similarity by using UPARSE v 7.0.1090 (Edgar, 2013). The most abundant sequence in the OTU was selected as the representative sequence. The taxonomic identity of each representative sequence was against the SILVA 132 reference databases (Quast et al., 2012) using the RDP Classifier method at a confidence threshold of 0.7 (Caporaso et al., 2012).

After the quality filter, 3,987,378 high-quality sequences in total were obtained from all ruminal-liquid samples (*n* = 40). Each sample contained reads ranging from 45,701 to 162,593. To standardize sequencing effort across samples, each sample was normalized to the number of 45,071 sequences. After normalization, the OTU diversity indices, rarefaction curves, and Good’s coverage were calculated by mothur (Schloss et al., 2009). For each sample, the coverage exceeds 0.991 (Supplementary Table S1), and the rarefaction curves tend to approach the saturation plateau (Supplementary Figure S1), suggesting sufficient coverage of the phylogenetic diversity after normalization.

### 2.5 Predicted ruminal bacterial functions and metabolic pathway based on 16S rRNA gene

The ruminal bacterial functions and metabolic pathways were inferred via the bioinformatics tool PICRUSt (Langille et al., 2013), based on 16S rRNA data. Paired-end assembling and quality-filtered reads were used for picking closed-reference OTUs against the Greengenes database (version 13.5) in QIIME 1.9.1 (Caporaso et al., 2012). The resulting OTU BIOM table was first normalized by the script normalize_by_copy_number.py. Next, Kyoto Encyclopedia of Genes and Genomes (KEGG) Orthologs were predicted using the script predict_metagenomes.py. Finally, KEGG pathways were summarized using the script categorize_by_function.py in the PICRUSt software (version1.1.3). We, thus, obtained the abundance table of KEGG pathways.

### 2.6 Co-occurrence network analysis

The ruminal bacterial co-occurrence network was constructed with all samples (H-sheep and T-sheep) by using the R package of microeco (Liu et al., 2021) with the SpiecEasi method (Kurtz et al., 2015) and then clustered highly correlated OTUs into the same ecological clusters (network modules) by igraph package (Csardi and Nepusz, 2006). The OTUs for co-occurrence network analysis were screened based on a relative abundance above 0.001 and presence in at least 90% of the samples (Xue et al., 2022). The relative abundance of each ecological cluster was calculated as the sum of the OTUs’ relative abundance in each module and presented with barplot with ggplot2 package in R software. The co-occurrence network and Spearman correlations between the microbial module eigengenes and rumen fermentation characteristics were visualized using Gephi software^1^ and the pheatmap package in R software, respectively.

### 2.7 Statistical analyses

The data were analyzed using the MIXED model procedures of SAS 9.2 (SAS Inst. Inc., Cary, NC). Dietary energy level and sheep breed were considered as fixed effects and experimental animals and periods as random effects. Polynomial contrasts were used to determine the effect of dietary energy level and the interaction with breed. Differences were considered significant at *p* < 0.05 and as a trend at 0.05 ≤ *p* < 0.10 (Zhou J. W. et al., 2019).

The constrained principal coordinate analysis (CPCoA) was done to visualize classical multidimensional scaling of Bray–Curtis distance matrices by using functions capscale and anova.cca of vegan package in R. Analysis of similarities (ANOSIM) was performed to test the differences in bacterial community composition among the different energy level diet treatments and between breeds by using function anosim of vegan package in R. Linear discriminant analysis effect size (LEfSe) analysis (Segata et al., 2011) was used to identify the specific bacteria taxa or KEGG pathway that were differentially altered among the different energy level diet treatment and between breeds.

## 3 Results

### 3.1 Rumen fermentation characteristics

The molar proportions of ruminal total VFA (average, 58.96 vs. 46.97 mmol/L, *p* = 0.002), acetate (average, 43.18 vs. 33.24 mmol/L, *p* = 0.005), butyrate (average, 4.70 vs. 3.86 mmol/L, *p* = 0.011), total branched-chain VFA (average, 1.88 vs. 1.52 mmol/L, *p* = 0.013), iso-butyrate (average, 0.70 vs. 0.56 mmol/L, *p* = 0.020), and iso-valerate (average, 0.85 vs. 0.66 mmol/L, *p* = 0.015) were higher in T-sheep than H-sheep. The molar proportions of ruminal valerate (average, 0.33 vs. 0.30 mmol/L, *p* = 0.076) and acetate-to-propionate ratio (average, 4.71 vs. 4.10, *p* = 0.067) showed a higher trend (*p* < 0.10) in T-sheep than H- sheep. There was no difference (*p* > 0.10) in ruminal pH and the molar proportion of propionate between T-sheep and H-sheep. With the increasing energy level of the diet, the molar proportion of valerate increased linearly (*p* < 0.05), and butyrate showed an increased trend (*p* < 0.10), whereas the acetate showed a decreased trend (*p* < 0.10) in both sheep breed. The other fermentation parameters and ruminal pH were similar among the different energy level diets. In addition, there was no interaction between sheep breed and dietary energy level for ruminal pH and fermentation parameters (*p* > 0.10) (Table 1).

**Table 1:** Ruminal pH and volatile fatty acid (VFA) production in H-sheep and T-sheep offered diets with different dietary energy levels.

Item	Breed	Dietary energy level, MJ/kg DM				SEM	*p*-value^*^			
		6.73	7.65	8.57	9.49		Breed	E-L	E-Q	E-C
pH	H	7.40	7.31	7.27	7.37	0.081	0.135	0.952	0.104	0.076
	T	7.26	7.03	7.32	7.22			0.456 ^x^	0.772 ^x^	0.030 ^x^
Total VFA (mmol/L)	H	49.36	50.12	42.36	46.04	3.441	0.002	0.161	0.668	0.183
	T	58.70	62.78	58.67	55.69			0.828 ^x^	0.296 ^x^	0.614 ^x^
Acetate (mmol/L)	H	35.72	34.78	29.95	32.52	2.920	0.005	0.068	0.706	0.222
	T	43.76	47.12	42.43	39.43			0.843^x^	0.191^x^	0.926^x^
Propionate (mmol/L)	H	8.43	9.57	7.66	7.76	0.658	0.106	0.276	0.530	0.139
	T	9.20	9.49	8.98	9.11			0.458^x^	0.632^x^	0.393^x^
Butyrate (mmol/L)	H	3.71	4.24	3.36	4.12	0.364	0.011	0.080	0.822	0.515
	T	4.07	4.40	5.25	5.10			0.133^x^	0.491^x^	0.058^x^
Total Branched-chain VFA (mmol/L)	H	1.51	1.54	1.39	1.64	0.160	0.013	0.122	0.715	0.830
	T	1.67	1.77	2.02	2.06			0.259^x^	0.525^x^	0.355^x^
Iso-butyrate (mmol/L)	H	0.58	0.57	0.51	0.58	0.067	0.020	0.438	0.873	0.886
	T	0.63	0.68	0.74	0.74			0.273^x^	0.489^x^	0.575^x^
Iso-valerate (mmol/L)	H	0.67	0.67	0.59	0.72	0.080	0.015	0.119	0.629	0.980
	T	0.75	0.78	0.93	0.94			0.212^x^	0.519^x^	0.299^x^
Valerate (mmol/L)	H	0.26	0.31	0.28	0.35	0.024	0.076	0.003	0.668	0.346
	T	0.29	0.31	0.35	0.38			0.667^x^	0.820^x^	0.209^x^
Acetate/Propionate	H	4.25	3.93	3.92	4.29	0.285	0.067	0.370	0.883	0.828
	T	4.77	4.96	4.74	4.35			0.284^x^	0.063^x^	0.889^x^

* E-L, linear effect of dietary energy; E-Q, quadratic effect of dietary energy; E-C, cubic effect of dietary energy.

x *p*-value for the interaction of dietary energy effect with the breed.

H, small-tailed Han sheep; T, Tibetan sheep.

### 3.2 Microbial community diversity and composition in rumen

**Figure 1:**
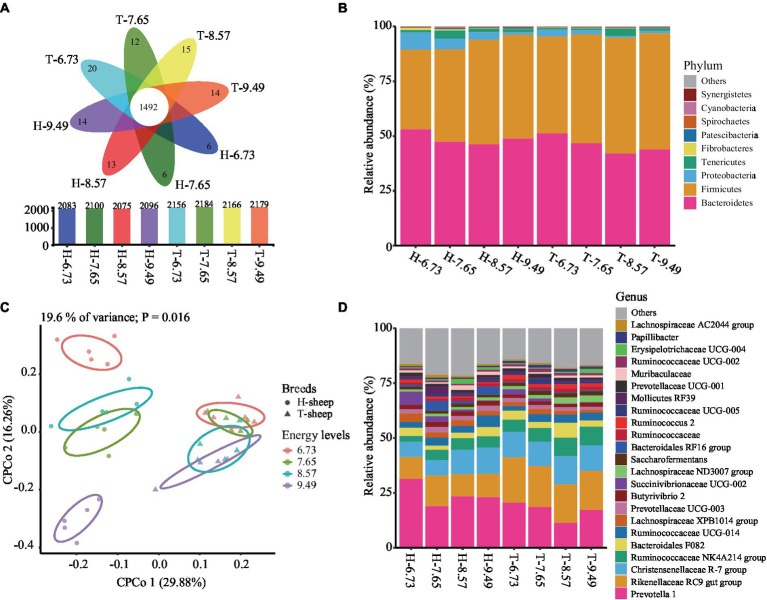
Effects of dietary energy levels (MJ ME/kg DM) on the Han sheep (H) and Tibetan sheep (T) rumen bacterial composition. **(A)** Flower plot showing the total, specific, and core OTUs between the sheep breed and among dietary energy levels. **(B)** Rumen bacterial relative abundance at phylum level between the sheep breed and among dietary energy levels. **(C)** Constrained principal coordinate analysis (CPCoA) of Bray–Curtis distance of ruminal microbiota effect by the dietary energy levels and breeds. **(D)** Rumen bacterial relative abundance at genus level between the sheep breed and among dietary energy levels (The numbers 6.73, 7.65, 8.57, and 9.49 indicate the diet energy levels of 6.73, 7.65, 8.57, and 9.49 MJ ME/kg DM, respectively).

After normalizing each sample to 45,071 sequences, a total of 2,639 OTUs were obtained. Among this, a total of 1,492 core OTUs were shared between the 8 treatment groups, accounting for 71.6, 71.0, 71.9, and 71.1% of each treatment group total OTUs in H-sheep for the 6.73, 7.65, 8.57, and 9.49 MJ/kg DM diets, and 69.2, 68.3, 68.9, and 68.5% of each treatment group total OTUs in T-sheep for the 6.73, 7.65, 8.57, and 9.49 MJ/kg DM diets, respectively (Figure 1A).

The number of ruminal bacteria observed species, ACE, and Shannon indices was greater (*p* < 0.05), while the Simpson indices were lower (*p* < 0.05) in T-sheep than H-sheep. These indices were both not affected by dietary energy level (*p* > 0.10), and interaction between sheep breed and dietary energy level (*p* > 0.10) (Table 2).

**Table 2:** Rumen microbial community richness and diversity in H-sheep and T-sheep offered diets with different dietary energy levels.

Indices	Breed	Dietary energy level (MJ/kg DM)				SEM	*p*-value^*^			
		6.73	7.65	8.57	9.49		Breed	E-L	E-Q	E-C
Observed species	H	1,277	1,296	1,272	1,287	28	0.007	0.821	0.618	0.259
	T	1,363	1,401	1,375	1,382			0.879^x^	0.708^x^	0.918^x^
ACE	H	1,622	1,665	1,603	1,644	32	0.014	0.547	0.663	0.210
	T	1710	1738	1745	1741			0.552^x^	0.686^x^	0.252^x^
Shannon	H	4.545	4.811	4.753	4.888	0.125	0.007	0.161	0.269	0.143
	T	4.931	5.227	5.037	5.058			0.331^x^	0.686^x^	0.821^x^
Simpson	H	0.058	0.035	0.046	0.030	0.008	0.044	0.307	0.368	0.121
	T	0.030	0.020	0.030	0.034			0.072^x^	0.696^x^	0.463^x^

* E-L, linear effect of dietary energy; E-Q, quadratic effect of dietary energy; E-C, cubic effect of dietary energy.

x *p*-value for the interaction of dietary energy effect with the breed.

H, small-tailed Han sheep; T, Tibetan sheep.

The bacterial OTUs were assigned to 21 different known phyla. The dominant bacteria phyla (relative abundance >1.00%) were Bacteroidetes, Firmicutes, Proteobacteria, and Tenericutes. In the H-sheep breed group, the average abundances of dominant phyla were 48.84, 43.34, 4.36, and 1.87%, respectively. In the T-sheep breed group, the average abundances of dominant phyla were 45.92, 49.95, 1.75, and 1.42%, respectively. These four phyla represent more than 98.10% of total bacterial reads (Figure 1B). The relative abundances of Bacteroidetes, Proteobacteria, and Tenericutes were similar between breeds (*p* > 0.10), while the relative abundances of Firmicutes were higher in T-sheep than H-sheep (*p* < 0.05). The relative abundance of Firmicutes showed a linear increase with a dietary energy level increase in both breeds (*p* < 0.05), while the relative abundance of Proteobacteria showed a linear decrease with a dietary energy level increase in both breeds (*p* < 0.05). The Firmicutes-to-Bacteroidetes ratio showed no significant difference in T-sheep and H-sheep (*p* > 0.10) but showed a linear increase with dietary energy level increased in both breeds (*p* < 0.05). There was no interaction between sheep breed and dietary energy level for ruminal bacterial abundance (*p* > 0.10) (Supplementary Table S2).

At the genus level, a total of 303 bacterial genera were identified. The dominant genus (relative abundance >1.00%) includes 23 genera, and the sum account is more than 80% of total bacteria reads (Figure 1D). These genera mainly belong to the phylum Bacteroidetes and Firmicutes.

In the H-sheep breed, the most abundance genus was *Prevotella* 1 and the second abundance genus was *Rikenellaceae* RC9 gut group, ranging from 22.67 to 31.00% and from 9.56 to 10.64%, respectively. In the T-sheep breed, the most abundance genus was *Rikenellaceae* RC9 gut group and the second abundance genus was *Prevotella* 1, ranging from 17.83 to 20.27% and from 16.87 to 20.16%, respectively (Figure 1D).

The relative abundance of *Lachnospiraceae* AC2044 group was higher (*p* < 0.05), and the relative abundance of *Prevotella* 1, *Lachnospiraceae* XPB1014 group, and *Bacteroidales* RF16 group tended to be greater (*p* < 0.10) in H-sheep than T-sheep, while the relative abundance of *Rikenellaceae* RC9 gut group, *Ruminococcaceae* NK4A214 group, *Saccharofermentans*, and *Papillibacter* were higher (*p* < 0.05) in T-sheep than H-sheep (Supplementary Table S3).

The relative abundance of *Christensenellaceae* R-7 group and *Ruminococcaceae* NK4A214 group showed a linear increase (*p* < 0.05) and *Ruminococcaceae* UCG-014 showed an increasing trend (*p* < 0.10) with the dietary energy level increased, while the abundance of *Succinivibrionaceae* UCG-002 showed a linear decrease (*p* < 0.05) and *Lachnospiraceae* XPB1014 group showed a decreased trend (*p* < 0.10) with the dietary energy level increased (Supplementary Table S3).

The CPCoA analysis revealed differences in the ruminal microbiota between the dietary energy levels and breeds, in which 19.6% of the total variance was explained by the dietary energy levels and breeds (*p* = 0.016, permutational multivariate analysis of variance). The percentage of interpretation in each axis corresponds to the fraction of the total variance by the projection. The CPCoA plot result showed that the ruminal bacterial profiles were distinct between the breeds and diets. The sheep breeds were separated by the first axis. The *p*-value of the ANOSIM result was 0.001, and the *R*-value was 0.251; however, the permdisper result had a *p*-value of 0.001, indicating no significant difference in rumen bacterial beta diversity among sheep breeds. Nevertheless, the ruminal bacterial profiles between different dietary energy levels were separated by the second axis but were actually similar among different dietary treatments in both T-sheep groups (ANOSIM, *p* = 0.912, *R* = −0.094) and H-sheep groups (ANOSIM, *p* = 0.979, *R* = −0.113) (Figure 1C).

### 3.3 The microbial co-occurrence network and its correlation with rumen fermentation characteristics

**Figure 2:**
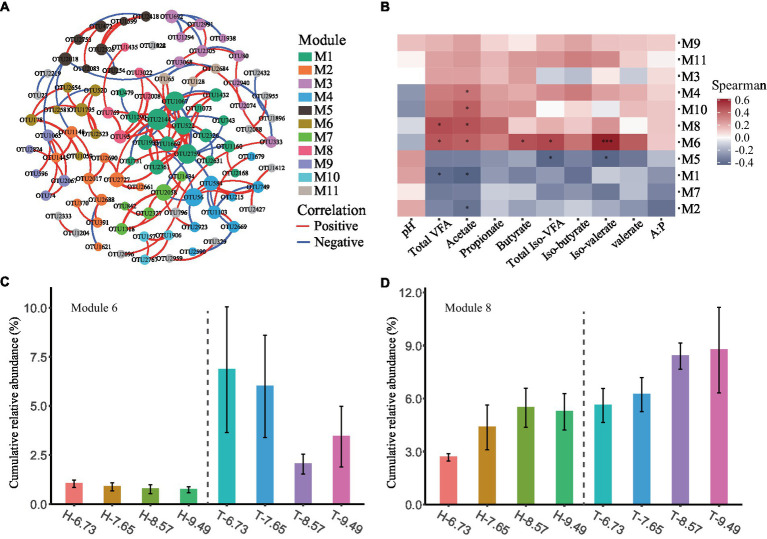
Co-occurrence patterns of ruminal microbes and their correlation with rumen fermentation characteristics. **(A)** The ruminal microbial co-occurrence network is shown, with nodes colored according to different modules. The size of each node is proportional to its degree. The red and blue lines represent positive and negative correlations, respectively. **(B)** The heatmap showed the Spearman correlation between the microbial module eigengenes and rumen fermentation characteristics. * and *** representing correlations of *p* < 0.05 and *p* < 0.001, respectively. **(C,D)** Barplot showing the cumulative relative abundance of microbial modules among different dietary energy levels and sheep breeds. Data are presented as means ± SE.

To investigate the changes in the ecological structure of rumen bacterial communities among sheep breeds and dietary energy levels, we conducted a co-occurrence network analysis to explore patterns of rumen bacterial distribution. After filtering the OTUs, a total of 172 rumen bacterial OTUs remained for co-occurrence network analysis. The co-occurrence network clustered 98 OTUs with significant correlation into 19 modules, with 11 modules containing at least three OTUs and the remaining modules having two OTUs (Figure 2A and Supplementary Table S4).

The M4, M6, M8, and M10 modules are positively correlated with various VFA concentrations in the rumen and negatively correlated with rumen pH. Conversely, the M1, M2, M5, and M7 modules are negatively correlated with various VFA concentrations in the rumen but positively correlated with rumen pH. The M9 and M11 modules are positively correlated with various VFAs and pH in the rumen (Figure 2B).

The cumulative relative abundance of modules varied among sheep breeds and dietary energy levels, with a higher cumulative relative abundance of M6 and M8 in T-sheep than in H-sheep (*p* < 0.05), and a higher trend for the cumulative relative abundance of M4 and M11 was observed in T-sheep than H-sheep (*p* < 0.10). The cumulative relative abundance of M7 showed a higher trend in H-sheep than T-sheep. In addition, the cumulative relative abundance of M7 and M8 increased with increasing dietary energy levels (*p* < 0.05) (Figures 2C,D and Supplementary Table S5).

Most of the rumen bacterial species in these modules are uncultured. At the genus level, M6 consists of six strains of Bacteroidetes and one strain of Firmicutes, including four strains of *Rikenellaceae* RC9 gut group, one strain each of *Bacteroidales* BS11 gut group, *Prevotellaceae* UCG-003, and *Ruminococcaceae* UCG-005. M8 consists of four strains of Firmicutes and one strain of Bacteroidetes, including two strains of *Ruminococcaceae* NK4A214 group, one strain each of *Rikenellaceae* RC9 gut group, *Ruminococcaceae* ge, and Unclassified genus of *Ruminococcaceae* (Supplementary Table S4).

### 3.4 Predicted ruminal bacterial functions and metabolic pathway by PICRUSt

**Figure 3:**
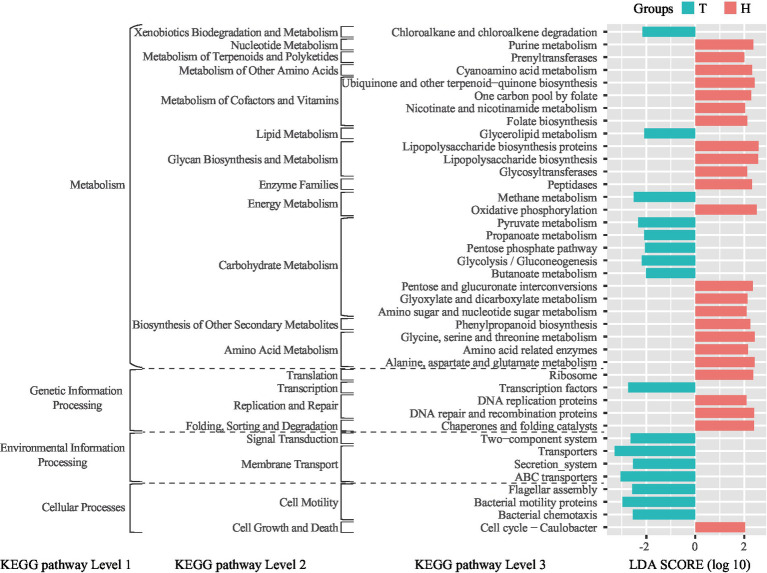
LEfSe analysis of linear discriminant analysis (LDA) plots metagenomic functional predictions of the KEGG pathway between the breeds (T: Tibetan sheep, H: small-tailed Han sheep).

The bioinformatics tool PICRUSt was used to predict the metabolic functional state of ruminal bacteria. Compared to the H-sheep, the T-sheep’s ruminal bacterial functions show significant enrichment in cell motility, membrane transport, signal transduction, transcription, lipid metabolism, and xenobiotics biodegradation and metabolism at KEGG pathway level 2. In contrast, the ruminal bacterial functions of H-sheep showed significant enrichment in cell growth and death, translation, replication and repair, folding, sorting and degradation, nucleotide metabolism, metabolism of terpenoids and polyketides, metabolism of other amino acids, metabolism of cofactors and vitamins, glycan biosynthesis and metabolism, enzyme families, biosynthesis of other secondary metabolites, and amino acid metabolism at KEGG pathway level 2. Among level 3 pathways within energy metabolism, the rumen bacteria of T-sheep exhibit an enriched function in methane metabolism, while the rumen bacteria of H-sheep demonstrate an enriched function in oxidative phosphorylation. Among the level 3 pathways within carbohydrate metabolism, T-sheep’s rumen bacteria are enriched in pyruvate metabolism, propanoate metabolism, pentose phosphate pathway, glycolysis/gluconeogenesis, and butanoate metabolism. On the other hand, H-sheep’s rumen bacteria are enriched in pentose and glucuronate interconversion, glyoxylate and dicarboxylate metabolism, amino sugar, and nucleotide sugar metabolism (Figure 3). In the H-sheep breed, there was only a fold change in the inositol phosphate metabolism pathway under the 7.65 MJ/kg DM dietary treatment. There were no differences observed in the KEGG pathway among dietary treatments in the T-sheep breed (Supplementary Figure S2).

## 4 Discussion

### 4.1 Effects of sheep species and dietary energy on rumen fermentation parameters

The fermentation of the rumen microbiome generates VFAs, which account for two-thirds of the carbon flow into ruminant metabolism and support normal growth, lactation, and reproduction in these animals. In the present study, T-sheep exhibited higher levels of total VFA, acetate, butyrate, iso-butyrate, and iso-valerate than H-sheep under the same dietary conditions. In addition, valerate levels and acetate-to-propionate ratio showed a higher trend in T-sheep. These results suggest that T-sheep have greater energy efficiency and are better adapted to the cold environment on the QTP than H-sheep. Similar findings have been reported for high-altitude ruminants compared to those at low altitudes (Zhang et al., 2016; Liu et al., 2022b).

In this study, the total VFA levels and pH remained relatively stable across the four dietary energy levels within each sheep breed. This finding is consistent with some previous studies that have reported similar results when animals are fed diets with different energy levels but the same amount of crude protein (Whelan et al., 2017; Wang et al., 2019). However, other studies have shown varying total VFAs among diets with different energy levels, which may be attributed to differences in crude protein content (Kang et al., 2012; Lu et al., 2019). The ruminal pH is influenced by the concentrations of VFAs and ammonia-N, as well as saliva secretions (Liu et al., 2022b). Changes in protein content within feed can lead to alterations in ammonia-N levels within the rumen, which subsequently affects VFA concentration to maintain relative stability of rumen pH.

The concentration of acetate exhibited a decreasing trend while that of butyrate showed an increasing trend and valerate increased linearly with the increase in dietary energy level. However, propionate remained constant despite the increase in dietary energy. These findings differ slightly from other studies which reported a decrease in acetate and an increase in butyrate, propionate, and valerate with an increase in dietary energy (Wang et al., 2019; Liu et al., 2022b). This discrepancy may be attributed to the fact that our study collected rumen fluid samples before morning feedings, while other studies collected them after feeding. During a night of microbiome fermentation and absorption of VFAs by the host, the concentration of propionate in the rumen remained constant.

Branched-chain VFAs are primarily produced through the fermentation of branched-chain amino acids (Wolin et al., 1997) and serve as essential metabolic substrates for the growth of fiber-degrading bacteria (Van Gylswyk, 1970). The concentration of total branched-chain VFAs is higher in T-sheep than H-sheep, which may explain why T-sheep have a greater concentration of acetate and can support more growth of fiber degradation bacteria.

### 4.2 Effects of sheep species and dietary energy on bacterial community composition

Although there were no significant changes in microbial alpha diversity with varying dietary energy levels, certain microbial taxa exhibited significant changes in relative abundance. The ruminal bacteria in T-sheep were found to be more abundant and diverse compared to those in H-sheep, which is consistent with our previous study that showed a higher concentration of microbial protein in the rumen of T-sheep (Zhou J. et al., 2019). This finding is also supported by Zhang et al. (2016), who reported a greater richness of ruminal bacteria in T-sheep than in ordinary sheep.

The phyla Bacteroidetes and Firmicutes were the predominant bacteria in the rumen of T-sheep and H-sheep, accounting for more than 90% of total bacterial sequences. These findings are consistent with previous studies that have identified Bacteroidetes and Firmicutes as dominant bacteria phyla under different dietary treatments in sheep rumens (Cui et al., 2019; Li R. et al., 2019), as well as in other ruminant rumens worldwide (Henderson et al., 2015). The Proteobacteria and Tenericutes were the third and fourth most abundant phyla in the rumen of sheep in this study. Bacteroidetes, Firmicutes, and Proteobacteria are copiotrophic taxa that exhibit potentially rapid growth rates and adaptability to high carbon availability (Gharechahi et al., 2021). Similar results were reported by Zhao et al. (2015) for steers’ rumen and Thoetkiattikul et al. (2013) for dairy cows’ rumen.

The metagene analysis of 4,941 rumen microorganisms showed that Bacteroides contained abundant polysaccharide utilization loci (PULz), among which 86% of the Bacteroides genome contained at least one PUL and most contained 52 PULs. Bacteroides proteins dedicated to carbohydrate-active enzyme (CAZyme) activity accounted for 5.7%, while Firmicutes accounted for 3.2% (Stewart et al., 2019). This finding indicates that Bacteroides possess a superior capacity for breaking down complex carbohydrates compared to Firmicutes, resulting in the dominance of the Bacteroides phylum in glycan-rich environments such as the gastrointestinal tracts of both humans and herbivores (Lapébie et al., 2019; McKee et al., 2021). Firmicutes are typically dominant in high-grain diets, and their relative abundance increases as the ratio of forage to concentrate increases (Faniyi et al., 2019). A higher ratio of Firmicutes to Bacteroidetes has been shown to affect energy extraction and is associated with increased fat accumulation and a higher average daily gain (Myer et al., 2015; Min et al., 2019). The present study found that the relative abundance of Firmicutes was higher in T-sheep than in H-sheep and increased linearly with the dietary energy level in both breeds. The high abundance of Firmicutes in T-sheep partially explains their higher energy harvesting efficiency compared to H-sheep. As dietary energy increased, the proportion of forage in the diet decreased while that of concentrate diets increased, resulting in a linear increase in Firmicutes abundance. Similarly, an increase in Firmicutes and a corresponding decrease in Bacteroidetes were observed with increasing dietary energy levels (Hu et al., 2020; Liu et al., 2022b). Proteobacteria also play crucial roles in the rumen, including the formation of biofilms and the fermentation of soluble carbohydrates (Pitta et al., 2016). The soluble carbohydrates increased with the dietary energy in the present study, which promoted the abundance of Proteobacteria.

In the H-sheep group, the *Prevotella* 1, *Bacteroidales* RF16 group, the Lachnospiraceae AC2044 group, and Lachnospiraceae XPB1014 group showed enrichment. The genus *Prevotella* 1 is primarily involved in the digestion of plant fibers (Ley, 2016) and protein degradation (Myer et al., 2015). It has been found to have a higher abundance under forage diets compared to concentrate diets. The genus *Bacteroidales* RF16 group has been reported to have a significantly higher abundance in cattle fed with forage compared to those fed with concentrate diets. In addition, it is negatively correlated with iso-butyrate and iso-valerate, suggesting that it may influence the production of these specific VFAs (Liu et al., 2019). The Lachnospiraceae AC2044 group and the *Lachnospiraceae* XPB1014 group have been reported to exhibit a positive correlation with milk fat content in dairy cows (Si et al., 2023). In the T-sheep group, the *Rikenellaceae* RC9 gut group, *Ruminococcaceae* NK4A214 group, *Saccharofermentans*, and *Papillibacter* showed enrichment. The *Rikenellaceae* RC9 gut group plays a role in degrading plant-derived polysaccharides in ruminants in QTP, potentially enhancing high-cellulose forage degradation (Xin et al., 2019; Lv et al., 2021), and also has been reported as positively correlated with average daily weigh gain (Daghio et al., 2021) and feed efficiency (Liu Y. et al., 2022). The abundance of *Ruminococcaceae* NK4A214 group was also reported as positively correlated with feed efficiency (Liu Y. et al., 2022). *Saccharofermentans* contributes to the breakdown of complex carbohydrates into simpler molecules that can be utilized by the host animal. *Papillibacter* is also a known butyrate producer, and increased abundances of *Papillibacter* result in a higher proportion of butyrate production (Xue Y. et al., 2020). The *Saccharofermentans* and *Papillibacter* were reported to contribute to the host’s adaptation to the harsh climate of high-altitude plateaus (Pan et al., 2024). The different microbial communities enriched in T-sheep and H-sheep exhibit varying digestion efficiencies of low-protein diets, which is one of the reasons for the disparities in their growth performance and environmental adaptability under poor diets.

The abundance of the *Christensenellaceae* R-7 group and the *Ruminococcaceae* NK4A214 group increased linearly, while there was an increasing trend in the abundance of *Ruminococcaceae* UCG-014 with dietary energy increased. Those three genera have been reported as positively correlated with feed efficiency (McLoughlin et al., 2020; Liu Y. et al., 2022). The *Ruminococcaceae* NK4A214 group is also reported to increase in response to a high-grain diet (Pan et al., 2017), suggesting a preference for non-structural polysaccharides and facilitated carbohydrate fermentation. The abundance of the *Succinivibrionaceae* UCG-002 decreased linearly, and there was a decreased trend in the *Lachnospiraceae* XPB1014 group as dietary energy increased. *Succinivibrionaceae* UCG-002, ureolytic bacteria, are known to enhance the diffusion of blood urea into the rumen (Honerlagen et al., 2023). The abundance of *Succinivibrionaceae* UCG-002 decreased linearly with an increase in dietary energy, which is one reason for the decrease in rumen urea. This finding aligns with a previous study that observed a decrease in rumen urea levels when dietary energy increased (Zhou J. et al., 2019). The *Lachnospiraceae* XPB1014 group decreased with an increase in dietary starch (Ricci et al., 2022), consistent with the findings of our research. The preferences of those genera for different metabolic substrates result in distinct patterns of change when the dietary energy increases.

### 4.3 Bacterial co-occurrence network modules vary with sheep species and dietary energy and correlate with fermentation parameters

The co-occurrence network analysis results indicate differential enrichment of certain modules (M4, M6, M8, and M11) among different sheep, and some modules (M7 and M8) exhibit linear changes as dietary energy increases. The abundance of specific modules shows differential enrichment among different sheep, indicating that each sheep has a distinct set of bacteria with enriched abundance. Furthermore, the abundance of specific modules demonstrates a linear change in response to dietary energy, suggesting a significant interaction between particular sets of bacteria and diet alterations. The modules M4, M6, M8, and M10 are positively correlated with various VFAs in the rumen and negatively correlated with pH, indicating that they produce VFAs. The abundance of modules M4, M6, and M8 is higher in T-sheep than in H-sheep, suggesting that T-sheep has a higher efficiency in VFA production. On the other hand, the modules M1, M2, M5, and M7 are negatively correlated with various VFAs in the rumen and positively correlated with pH, indicating that they consume VFAs. Modules M9 and M11 are positively correlated with various VFAs and pH levels, suggesting they enhance VFA production while maintaining a healthy ruminal pH level. This is crucial for improving ruminant productivity and health. The differences in abundance of these modules result in variations in ruminal metabolism among different sheep breeds and influence their response to various energy diets. However, most of the bacteria in these modules are unclassified at the species level, which limits further research on them. At the family level, bacteria in these modules mainly belong to Christensenellaceae, Lachnospiraceae, Prevotellaceae, Rikenellaceae, and Ruminococcaceae. These groups should be paid attention to during subsequent isolation and culture of rumen bacteria as they contain potentially efficient strains that can be used as bacterial agents to regulate rumen metabolism and production in ruminants (Ishaq et al., 2016).

### 4.4 Functions of rumen bacteria vary with sheep species and dietary energy

Based on the PICRUSt analysis of rumen bacteria, we found that different sheep breeds exhibit distinct rumen metabolic characteristics under the same dietary treatment. However, changes in dietary energy levels do not significantly alter the rumen metabolic characteristics. The rumen bacteria of the T-sheep exhibited a stronger preference for energy-related utilization and processing of environmental information. For example, the breakdown of glycerolipids in glycerolipid metabolism releases glycerol and fatty acids, which can be further metabolized to produce energy (Xue M.-Y. et al., 2020) and affect the fatty acid profile of ruminant milk and meat (Jarvis and Moore, 2010). Pyruvate metabolism is a central part of the energy production process in rumen bacteria. In the absence of oxygen, rumen bacteria ferment pyruvate to produce volatile fatty acids (VFAs) such as acetate, propionate, and butyrate, which serve as important energy sources for the host animal (Ungerfeld, 2020). Propanoate metabolism is an essential process in rumen bacteria, contributing to energy production and the synthesis of various metabolites. Propanoate serves as a crucial intermediate in energy metabolism and a necessary substrate for gluconeogenesis in ruminants. It can be converted into glucose in the liver, providing energy for the animal. The pentose phosphate pathway plays a crucial role in the oxidation of glucose and the production of reducing equivalents (NADPH) and ribose-5-phosphate, which are essential for biosynthetic processes and antioxidant defense, and as precursors for nucleotide synthesis. The pentose phosphate pathway contributes to energy production, biosynthesis, and maintaining cellular redox homeostasis in rumen bacteria (Anderson et al., 2017). Glycolysis is the process of breaking down glucose into pyruvate, yielding ATP and NADH as energy sources. In the rumen, glycolysis plays a crucial role in providing energy under anaerobic conditions. Gluconeogenesis is the synthesis of glucose from non-carbohydrate sources such as lactate, amino acids, and glycerol. This pathway is particularly important in ruminants as it allows for the conversion of propionate, one of the main volatile fatty acids produced in the rumen, into glucose which serves as a critical energy source for the host animal. Butyrate metabolism is a critical process in rumen bacteria. Butyrate can be utilized by the host animal as an energy substrate and is essential for maintaining the health of the rumen epithelium (Niwińska et al., 2017). In addition, it possesses anti-inflammatory properties and supports gut integrity (Pryde et al., 2002). These functional characteristics of rumen bacteria can help T-sheep better adapt to the harsh environment on the Qinghai–Tibet Plateau and are also a result of co-evolution between the rumen bacteria and the host.

The rumen bacteria of the H-sheep exhibited a stronger preference for nucleotide metabolism, metabolism of terpenoids and polyketides, metabolism of other amino acids, metabolism of cofactors and vitamins, biosynthesis of other secondary metabolites, amino acid metabolism, replication and repair, folding, sorting, and degradation at the level 2 of the KEGG pathway. These pathways are closely related to protein synthesis, and they are also part of the reason for the high reproduction rate, high slaughter rate, and high meat quality of H-sheep because these processes require a large amount of protein. However, these characteristics are not well expressed under conditions of low-protein feeding. Nevertheless, the functional differences between rumen bacteria in H-sheep and T-sheep based on the prediction from 16S data do not fully reflect the function of ruminal bacteria. Metagenomic and meta-transcriptomic approaches were used to gain a better understanding of functional differences in further studies. Although previous studies have shown that T-sheep can utilize nitrogen more effectively under low-protein diet conditions (Zhou J. W. et al., 2019), our study demonstrates that the ruminal microbial function of H-sheep is more biased toward protein metabolism-related functions. Subsequent research can be conducted when there is sufficient dietary protein and energy, which will confirm that the total nitrogen utilization of H-sheep is higher than that of T-sheep.

## 5 Conclusion

The rumen total VFAs, acetate, butyrate, total branched-chain VFAs, iso-butyrate, and iso-valerate were higher in T-sheep than H-sheep. Valerate increased linearly, and butyrate showed an increasing trend, whereas acetate showed a decreasing trend with an increase in dietary energy. The alpha diversity of ruminal bacteria was not affected by diet energy, but it differed between sheep breeds. Observer species, ACE, and Shannon indices showed higher values in the T-sheep than H-sheep, while the Simpson index showed lower values. The beta diversity of ruminal bacteria is not affected by sheep breeds or dietary energy, indicating that the rumen bacterial community is similar between the different sheep breeds and levels of dietary energy. The phyla Bacteroidetes and Firmicutes predominate in both sheep rumens, with the relative abundance of Firmicutes being higher in T-sheep than H-sheep. The two most abundant genera in the rumen are *Prevotella* 1 and *Rikenellaceae* RC9 gut group. *Prevotella* 1 is the most prevalent bacterial genus in the rumen of H-sheep, while *Rikenellaceae* RC9 gut group dominates in the rumen of T-sheep. The different microbial communities enriched in T-sheep and H-sheep exhibit varying digestion efficiencies of low-protein diets, which is one of the reasons for the disparities in their growth performance and environmental adaptability under poor diets. Microbial co-occurrence network analysis reveals that variations in rumen fermentation characteristics result from differences in module abundance, with a higher abundance of VFA-producing modules observed in the rumen of T-sheep. Microbial function prediction analysis showed that dietary energy had no significant impact on the functional composition of rumen bacteria. There were differences in the functions of rumen bacteria between sheep breeds, with T-sheep showing a greater emphasis on energy metabolism-related functions, while H-sheep showed a greater emphasis on protein metabolism-related functions. These findings provide evidence that the special rumen microbial community helps the T-sheep to obtain energy more efficiently from low-protein and low-energy diets, allowing them to survive in the extreme environment of the Qinghai–Tibet Plateau. However, under such harsh conditions, the H-sheep cannot demonstrate its ability to emphasize protein metabolism-related functions. Subsequent research can be conducted when there is sufficient dietary protein and energy, which will confirm that the total nitrogen utilization of H-sheep is higher than that of T-sheep.

## Data availability statement

The raw sequence data of the bacterial 16S rRNA gene in all rumen fluid were deposited in the NCBI Sequence Read Archive (SRA) under the BioProject number PRJNA534195.

## Ethics statement

The animal study was approved by all procedures involving the use of animals were approved by the Animal Ethics Committee at the School of Life Sciences of Lanzhou University (SCXK Gan 20140215). The study was conducted in accordance with the local legislation and institutional requirements.

## Author contributions

XW: Data curation, Formal analysis, Methodology, Software, Visualization, Writing – original draft, Writing – review & editing. GZ: Visualization, Writing – review & editing. WZ: Writing – review & editing. JZ: Conceptualization, Investigation, Resources, Writing – review & editing. HC: Investigation, Resources, Writing – review & editing. GY: Conceptualization, Project administration, Supervision, Validation, Writing – review & editing. GL: Project administration, Supervision, Validation, Writing – review & editing.

## Footnotes

^1^https://gephi.org
