# Supplementary material for: Rumen microbiota helps Tibetan sheep obtain energy more efficiently to survive in the extreme environment of the Qinghai–Tibet Plateau
Source: Front Microbiol. 2024 Jul 24;15:1431063. doi: 10.3389/fmicb.2024.1431063 (PMC11303141; doi:10.3389/fmicb.2024.1431063)
Supplement: Supplementary file 1 [file Table_1.docx]

Table S1. Good’s coverage of OTUs clustered at 97% similarity level across different samples

| Dietary energy level (MJ/kg DM) | Breed | Samples | | | | |
| --- | --- | --- | --- | --- | --- | --- |
|  |  | 1 | 2 | 3 | 4 | 5 |
| 6.73 | H | 0.992 | 0.992 | 0.992 | 0.992 | 0.993 |
|  | T | 0.992 | 0.992 | 0.991 | 0.992 | 0.992 |
| 7.65 | H | 0.992 | 0.992 | 0.993 | 0.992 | 0.993 |
|  | T | 0.992 | 0.991 | 0.992 | 0.992 | 0.993 |
| 8.57 | H | 0.992 | 0.992 | 0.992 | 0.992 | 0.992 |
|  | T | 0.992 | 0.992 | 0.992 | 0.993 | 0.992 |
| 9.49 | H | 0.992 | 0.992 | 0.993 | 0.992 | 0.992 |
|  | T | 0.991 | 0.993 | 0.992 | 0.992 | 0.992 |

H, small-tailed Han sheep; T, Tibetan sheep.

Table S2. Composition of dominate ruminal bacterial communities at phylum level (relative abundance > 1.00%)

| Phylum | Breed | Dietary energy level (MJ/kg DM) | | | | SEM | *P*-value^*^ | | | |
| --- | --- | --- | --- | --- | --- | --- | --- | --- | --- | --- |
|  |  | 6.73 | 7.65 | 8.57 | 9.49 |  | Breed | E-L | E-Q | E-C |
| Bacteroidetes | H | 52.09 | 47.62 | 47.16 | 48.50 | 4.356 | 0.431 | 0.197 | 0.416 | 0.908 |
|  | T | 50.22 | 46.92 | 42.94 | 43.58 |  |  | 0.629 ^x^ | 0.973 ^x^ | 0.774 ^x^ |
| Firmicutes | H | 36.69 | 41.85 | 46.88 | 48.30 | 3.641 | 0.037 | 0.006 | 0.523 | 0.919 |
|  | T | 44.52 | 49.32 | 52.07 | 53.88 |  |  | 0.694 ^x^ | 0.942 ^x^ | 0.840 ^x^ |
| Proteobacteria | H | 8.21 | 4.81 | 3.53 | 0.89 | 2.072 | 0.140 | 0.019 | 0.676 | 0.836 |
|  | T | 3.55 | 1.93 | 0.74 | 0.79 |  |  | 0.288 ^x^ | 0.872 ^x^ | 0.737 ^x^ |
| Tenericutes | H | 1.17 | 3.83 | 1.38 | 1.11 | 1.274 | 0.626 | 0.921 | 0.146 | 0.958 |
|  | T | 0.84 | 0.94 | 3.18 | 0.70 |  |  | 0.584 ^x^ | 0.923 ^x^ | 0.091 ^x^ |
| Firmicutes / Bacteroidetes ratio | H | 0.77 | 0.91 | 1.14 | 1.03 | 0.187 | 0.220 | 0.040 | 0.294 | 0.470 |
|  | T | 0.90 | 1.11 | 1.38 | 1.29 |  |  | 0.696 ^x^ | 0.913 ^x^ | 0.990 ^x^ |

^*^E-L = linear effect of dietary energy; E-Q = quadratic effect of dietary energy; E-C = cubic effect of dietary energy.

^x^*P*-value for the interaction of dietary energy effect with the breed.

H, small-tailed Han sheep; T, Tibetan sheep.

Table S3. Composition of dominate ruminal bacterial communities at genus level (relative abundance > 1.00%)

| Phylum | Genus | Breed | Dietary energy level (MJ/kg DM) | | | | SEM | *P*-value^*^ | | | |
| --- | --- | --- | --- | --- | --- | --- | --- | --- | --- | --- | --- |
|  |  |  | 6.73 | 7.65 | 8.57 | 9.49 |  | Breed | E-L | E-Q | E-C |
| Bacteroidetes | *Prevotella* 1 | H | 31.00 | 19.10 | 23.66 | 22.67 | 4.623 | 0.055 | 0.219 | 0.196 | 0.901 |
|  |  | T | 20.16 | 18.73 | 11.54 | 16.87 |  |  | 0.909 ^x^ | 0.753 ^x^ | 0.180 ^x^ |
| Bacteroidetes | *Rikenellaceae* RC9 gut group | H | 9.56 | 14.24 | 10.32 | 10.64 | 2.769 | 0.005 | 0.615 | 0.700 | 0.453 |
|  |  | T | 20.27 | 18.94 | 17.89 | 17.83 |  |  | 0.662 ^x^ | 0.478 ^x^ | 0.493 ^x^ |
| Firmicutes | *Christensenellaceae* R-7 group | H | 6.82 | 6.93 | 10.71 | 12.74 | 1.946 | 0.105 | 0.049 | 0.692 | 0.473 |
|  |  | T | 11.40 | 10.94 | 12.49 | 12.35 |  |  | 0.175 ^x^ | 0.773 ^x^ | 0.819 ^x^ |
| Firmicutes | *Ruminococcaceae* NK4A214 group | H | 2.69 | 4.67 | 5.12 | 5.22 | 1.261 | 0.015 | 0.032 | 0.583 | 0.856 |
|  |  | T | 5.70 | 6.28 | 8.13 | 8.59 |  |  | 0.757 ^x^ | 0.623 ^x^ | 0.632 ^x^ |
| Bacteroidetes | *Bacteroidales* F082 | H | 3.77 | 1.82 | 2.72 | 3.99 | 1.982 | 0.478 | 0.903 | 0.964 | 0.195 |
|  |  | T | 4.11 | 2.69 | 7.04 | 2.66 |  |  | 0.901 ^x^ | 0.276 ^x^ | 0.343 ^x^ |
| Firmicutes | *Ruminococcaceae* UCG-014 | H | 2.65 | 3.62 | 3.20 | 5.16 | 0.917 | 0.500 | 0.064 | 0.763 | 0.480 |
|  |  | T | 2.07 | 3.50 | 3.80 | 3.44 |  |  | 0.624 ^x^ | 0.295 ^x^ | 0.570 ^x^ |
| Firmicutes | *Lachnospiraceae* XPB1014 group | H | 4.00 | 2.34 | 2.65 | 2.09 | 0.626 | 0.069 | 0.073 | 0.828 | 0.707 |
|  |  | T | 1.93 | 2.33 | 1.72 | 1.41 |  |  | 0.412 ^x^ | 0.316 ^x^ | 0.304 ^x^ |
| Bacteroidetes | *Prevotellaceae* UCG-003 | H | 1.95 | 2.45 | 1.93 | 2.46 | 0.525 | 0.318 | 0.795 | 0.907 | 0.508 |
|  |  | T | 1.88 | 1.76 | 1.64 | 1.34 |  |  | 0.333 ^x^ | 0.861 ^x^ | 0.425 ^x^ |
| Firmicutes | *Butyrivibrio* 2 | H | 1.85 | 2.32 | 2.10 | 2.20 | 0.346 | 0.181 | 0.345 | 0.903 | 0.364 |
|  |  | T | 1.53 | 1.67 | 1.53 | 1.93 |  |  | 0.916 ^x^ | 0.478 ^x^ | 0.927 ^x^ |
| Proteobacteria | *Succinivibrionaceae* UCG-002 | H | 6.38 | 2.43 | 0.71 | 0.18 | 1.728 | 0.284 | 0.015 | 0.329 | 0.993 |
|  |  | T | 2.69 | 1.23 | 0.06 | 0.12 |  |  | 0.303 ^x^ | 0.699 ^x^ | 0.857 ^x^ |
| Firmicutes | *Lachnospiraceae* ND3007 group | H | 1.12 | 1.28 | 1.05 | 1.34 | 0.826 | 0.110 | 0.272 | 0.899 | 0.878 |
|  |  | T | 1.54 | 1.66 | 2.74 | 3.03 |  |  | 0.335 ^x^ | 0.989 ^x^ | 0.609 ^x^ |
| Firmicutes | *Saccharofermentans* | H | 1.12 | 1.21 | 1.37 | 1.25 | 0.211 | 0.005 | 0.175 | 0.499 | 0.772 |
|  |  | T | 1.54 | 1.92 | 1.83 | 2.01 |  |  | 0.561 ^x^ | 0.978 ^x^ | 0.403 ^x^ |
| Bacteroidetes | *Bacteroidales* RF16 group | H | 0.95 | 4.48 | 1.21 | 3.65 | 1.292 | 0.096 | 0.478 | 0.755 | 0.123 |
|  |  | T | 0.39 | 0.35 | 0.54 | 0.53 |  |  | 0.575 ^x^ | 0.739 ^x^ | 0.094 ^x^ |
| Firmicutes | *Ruminococcaceae* | H | 1.10 | 1.23 | 1.59 | 1.45 | 0.229 | 0.619 | 0.102 | 0.246 | 0.288 |
|  |  | T | 1.29 | 1.43 | 1.68 | 1.43 |  |  | 0.552 ^x^ | 0.817 ^x^ | 0.911 ^x^ |
| Firmicutes | *Ruminococcus* 2 | H | 1.36 | 0.80 | 0.66 | 0.72 | 0.532 | 0.172 | 0.355 | 0.417 | 0.380 |
|  |  | T | 1.98 | 1.21 | 1.85 | 1.53 |  |  | 0.643 ^x^ | 0.898 ^x^ | 0.455 ^x^ |
| Firmicutes | *Ruminococcaceae* UCG-005 | H | 0.52 | 1.34 | 0.88 | 0.95 | 0.544 | 0.277 | 0.760 | 0.202 | 0.058 |
|  |  | T | 1.19 | 2.55 | 0.94 | 1.12 |  |  | 0.413 ^x^ | 0.768 ^x^ | 0.370 ^x^ |
| Tenericutes | *Mollicutes* RF39 | H | 1.02 | 3.59 | 0.99 | 0.93 | 0.963 | 0.261 | 0.593 | 0.240 | 0.265 |
|  |  | T | 0.68 | 0.76 | 0.99 | 0.48 |  |  | 0.666 ^x^ | 0.445 ^x^ | 0.155 ^x^ |
| Bacteroidetes | *Prevotellaceae* UCG-001 | H | 1.58 | 1.08 | 1.07 | 1.15 | 0.300 | 0.746 | 0.240 | 0.940 | 0.580 |
|  |  | T | 1.01 | 1.53 | 1.05 | 0.92 |  |  | 0.730 ^x^ | 0.110 ^x^ | 0.295 ^x^ |
| Bacteroidetes | *Muribaculaceae* | H | 0.87 | 0.70 | 2.23 | 1.46 | 0.651 | 0.474 | 0.275 | 0.617 | 0.223 |
|  |  | T | 0.72 | 0.81 | 1.17 | 0.96 |  |  | 0.573 ^x^ | 0.862 ^x^ | 0.426 ^x^ |
| Firmicutes | *Lachnospiraceae* Other | H | 0.95 | 2.95 | 0.80 | 0.671 | 0.691 | 0.309 | 0.334 | 0.259 | 0.141 |
|  |  | T | 0.92 | 0.92 | 0.68 | 0.579 |  |  | 0.685 ^x^ | 0.292 ^x^ | 0.182 ^x^ |
| Firmicutes | *Ruminococcaceae* UCG-002 | H | 0.91 | 0.86 | 0.70 | 0.88 | 0.307 | 0.148 | 0.705 | 0.541 | 0.288 |
|  |  | T | 0.98 | 1.66 | 1.10 | 1.00 |  |  | 0.900 ^x^ | 0.252 ^x^ | 0.529 ^x^ |
| Firmicutes | *Erysipelotrichaceae* UCG-004 | H | 0.51 | 0.44 | 2.34 | 1.17 | 0.771 | 0.695 | 0.081 | 0.890 | 0.318 |
|  |  | T | 0.50 | 0.32 | 0.71 | 1.93 |  |  | 0.863 ^x^ | 0.235 ^x^ | 0.264 ^x^ |
| Firmicutes | *Papillibacter* | H | 0.52 | 0.68 | 0.61 | 0.61 | 0.124 | <0.001 | 0.547 | 0.284 | 0.532 |
|  |  | T | 1.05 | 1.24 | 1.21 | 1.16 |  |  | 0.879 ^x^ | 0.834 ^x^ | 0.919 ^x^ |
| Firmicutes | *Lachnospiraceae* AC2044 group | H | 1.14 | 1.30 | 0.79 | 0.86 | 0.196 | 0.035 | 0.123 | 0.908 | 0.174 |
|  |  | T | 0.75 | 0.78 | 0.56 | 0.61 |  |  | 0.564 ^x^ | 0.835 ^x^ | 0.551 ^x^ |

^*^E-L = linear effect of dietary energy; E-Q = quadratic effect of dietary energy; E-C = cubic effect of dietary energy.

^x^*P*-value for the interaction of dietary energy effect with the breed.

H, small-tailed Han sheep; T, Tibetan sheep.

Table S4. Node attribute in the co-occurrence network

| **Node** | **degree** | **Between-ness** | **module** | **Zi** | **Ci** | **taxa_roles** | **Kingdom** | **Phylum** | **Class** | **Order** | **Family** | **Genus** | **Species** |
| --- | --- | --- | --- | --- | --- | --- | --- | --- | --- | --- | --- | --- | --- |
| OTU1993 | 5 | 1070 | M1 | 0.351 | 0.000 | Peripheral nodes | Bacteria | Bacteroidetes | Bacteroidia | Bacteroidales | Prevotellaceae | g__Prevotella_1 | s__ |
| OTU522 | 7 | 21 | M1 | 1.097 | 0.000 | Peripheral nodes | Bacteria | Bacteroidetes | Bacteroidia | Bacteroidales | Prevotellaceae | g__Prevotella_1 | s__uncultured_bacterium |
| OTU2326 | 4 | 68 | M1 | -0.022 | 0.000 | Peripheral nodes | Bacteria | Bacteroidetes | Bacteroidia | Bacteroidales | Prevotellaceae | g__Prevotella_1 | s__uncultured_bacterium |
| OTU743 | 2 | 1071 | M1 | -0.768 | 0.000 | Peripheral nodes | Bacteria | Bacteroidetes | Bacteroidia | Bacteroidales | Rikenellaceae | g__Rikenellaceae_RC9_gut_group | s__uncultured_bacterium |
| OTU1073 | 1 | 0 | M1 | -1.141 | 0.500 | Peripheral nodes | Bacteria | Bacteroidetes | Bacteroidia | Bacteroidales | Rikenellaceae | g__Rikenellaceae_RC9_gut_group | s__uncultured_rumen_bacterium |
| OTU1290 | 3 | 67 | M1 | -0.395 | 0.444 | Peripheral nodes | Bacteria | Fibrobacteres | Fibrobacteria | Fibrobacterales | Fibrobacteraceae | g__Fibrobacter | s__ |
| OTU731 | 1 | 0 | M1 | -1.141 | 0.000 | Peripheral nodes | Bacteria | Firmicutes | Clostridia | Clostridiales | Christensenellaceae | g__Christensenellaceae_R-7_group | s__uncultured_rumen_bacterium |
| OTU479 | 1 | 0 | M1 | -1.141 | 0.000 | Peripheral nodes | Bacteria | Firmicutes | Clostridia | Clostridiales | Christensenellaceae | g__Christensenellaceae_R-7_group | s__uncultured_sludge_bacterium |
| OTU2361 | 5 | 6 | M1 | 0.351 | 0.000 | Peripheral nodes | Bacteria | Firmicutes | Clostridia | Clostridiales | Lachnospiraceae | g__ | s__ |
| OTU2631 | 2 | 0 | M1 | -0.768 | 0.000 | Peripheral nodes | Bacteria | Firmicutes | Clostridia | Clostridiales | Ruminococcaceae | g__ | s__ |
| OTU1067 | 8 | 15 | M1 | 1.470 | 0.000 | Peripheral nodes | Bacteria | Firmicutes | Clostridia | Clostridiales | Ruminococcaceae | g__Papillibacter | s__uncultured_rumen_bacterium |
| OTU1669 | 5 | 372 | M1 | 0.351 | 0.000 | Peripheral nodes | Bacteria | Firmicutes | Clostridia | Clostridiales | Ruminococcaceae | g__Papillibacter | s__uncultured_rumen_bacterium |
| OTU2168 | 1 | 0 | M1 | -1.141 | 0.000 | Peripheral nodes | Bacteria | Firmicutes | Clostridia | Clostridiales | Ruminococcaceae | g__Ruminococcaceae_UCG-014 | s__unidentified |
| OTU1160 | 3 | 187 | M1 | -0.395 | 0.000 | Peripheral nodes | Bacteria | Firmicutes | Clostridia | Clostridiales | Ruminococcaceae | g__Ruminococcus_1 | s__Ruminococcus_flavefaciens |
| OTU2144 | 9 | 397 | M1 | 1.844 | 0.000 | Peripheral nodes | Bacteria | Proteobacteria | Gammaproteobacteria | Aeromonadales | Succinivibrionaceae | g__Ruminobacter | s__uncultured_rumen_bacterium |
| OTU2759 | 8 | 1114 | M1 | 1.470 | 0.000 | Peripheral nodes | Bacteria | Proteobacteria | Gammaproteobacteria | Aeromonadales | Succinivibrionaceae | g__Succinivibrio | s__uncultured_rumen_bacterium |
| OTU1432 | 4 | 0 | M1 | -0.022 | 0.000 | Peripheral nodes | Bacteria | Proteobacteria | Gammaproteobacteria | Aeromonadales | Succinivibrionaceae | g__Succinivibrionaceae_UCG-002 | s__uncultured_rumen_bacterium |
| OTU1148 | 3 | 987 | M2 | 0.559 | 0.000 | Peripheral nodes | Bacteria | Bacteroidetes | Bacteroidia | Bacteroidales | Prevotellaceae | g__Prevotella_1 | s__uncultured_rumen_bacterium |
| OTU1621 | 1 | 0 | M2 | -1.039 | 0.444 | Peripheral nodes | Bacteria | Bacteroidetes | Bacteroidia | Bacteroidales | Prevotellaceae | g__Prevotella_1 | s__uncultured_rumen_bacterium |
| OTU391 | 2 | 67 | M2 | -0.240 | 0.000 | Peripheral nodes | Bacteria | Firmicutes | Clostridia | Clostridiales | Lachnospiraceae | g__Butyrivibrio_2 | s__ |
| OTU2688 | 3 | 197 | M2 | 0.559 | 0.000 | Peripheral nodes | Bacteria | Firmicutes | Clostridia | Clostridiales | Lachnospiraceae | g__Butyrivibrio_2 | s__ |
| OTU2661 | 1 | 0 | M2 | -1.039 | 0.000 | Peripheral nodes | Bacteria | Firmicutes | Clostridia | Clostridiales | Lachnospiraceae | g__Lachnospiraceae_AC2044_group | s__uncultured_bacterium |
| OTU1445 | 2 | 0 | M2 | -0.240 | 0.444 | Peripheral nodes | Bacteria | Firmicutes | Clostridia | Clostridiales | Lachnospiraceae | g__Lachnospiraceae_AC2044_group | s__uncultured_rumen_bacterium |
| OTU370 | 1 | 0 | M2 | -1.039 | 0.000 | Peripheral nodes | Bacteria | Firmicutes | Clostridia | Clostridiales | Lachnospiraceae | g__Lachnospiraceae_XPB1014_group | s__uncultured_bacterium |
| OTU2690 | 2 | 1107 | M2 | -0.240 | 0.219 | Peripheral nodes | Bacteria | Firmicutes | Clostridia | Clostridiales | Lachnospiraceae | g__Pseudobutyrivibrio | s__uncultured_rumen_bacterium |
| OTU2017 | 3 | 1180 | M2 | 0.559 | 0.640 | Connectors | Bacteria | Firmicutes | Clostridia | Clostridiales | Lachnospiraceae | g__uncultured | s__Lachnospiraceae bacterium CG57 |
| OTU2727 | 5 | 1381 | M2 | 2.157 | 0.000 | Peripheral nodes | Bacteria | Firmicutes | Clostridia | Clostridiales | Ruminococcaceae | g__Ruminococcaceae_UCG-014 | s__uncultured_rumen_bacterium |
| OTU692 | 4 | 67 | M3 | 1.488 | 0.000 | Peripheral nodes | Bacteria | Bacteroidetes | Bacteroidia | Bacteroidales | Prevotellaceae | g__Prevotella_1 | s__uncultured_bacterium |
| OTU2940 | 1 | 0 | M3 | -1.302 | 0.500 | Peripheral nodes | Bacteria | Bacteroidetes | Bacteroidia | Bacteroidales | Rikenellaceae | g__Rikenellaceae_RC9_gut_group | s__uncultured_rumen_bacterium |
| OTU3068 | 3 | 637 | M3 | 0.558 | 0.000 | Peripheral nodes | Bacteria | Bacteroidetes | Bacteroidia | Bacteroidales | Rikenellaceae | g__Rikenellaceae_RC9_gut_group | s__uncultured_rumen_bacterium |
| OTU1938 | 1 | 0 | M3 | -1.302 | 0.000 | Peripheral nodes | Bacteria | Bacteroidetes | Bacteroidia | Bacteroidales | Rikenellaceae | g__U29-B03 | s__uncultured_rumen_bacterium |
| OTU1294 | 2 | 0 | M3 | -0.372 | 0.625 | Connectors | Bacteria | Firmicutes | Clostridia | Clostridiales | Christensenellaceae | g__Christensenellaceae_R-7_group | s__ |
| OTU2991 | 3 | 67 | M3 | 0.558 | 0.000 | Peripheral nodes | Bacteria | Firmicutes | Clostridia | Clostridiales | Christensenellaceae | g__Christensenellaceae_R-7_group | s__ |
| OTU2305 | 3 | 256 | M3 | 0.558 | 0.000 | Peripheral nodes | Bacteria | Firmicutes | Clostridia | Clostridiales | Christensenellaceae | g__Christensenellaceae_R-7_group | s__uncultured Clostridiales bacterium |
| OTU2074 | 1 | 0 | M3 | -1.302 | 0.000 | Peripheral nodes | Bacteria | Firmicutes | Clostridia | Clostridiales | Lachnospiraceae | g__Butyrivibrio_2 | s__Butyrivibrio_fibrisolvens |
| OTU333 | 3 | 197 | M3 | 0.558 | 0.000 | Peripheral nodes | Bacteria | Firmicutes | Clostridia | Clostridiales | Lachnospiraceae | g__probable_genus_10 | s__uncultured_bacterium |
| OTU80 | 3 | 551 | M3 | 0.558 | 0.000 | Peripheral nodes | Bacteria | Firmicutes | Clostridia | Clostridiales | Ruminococcaceae | g__Ruminococcaceae_ge | s__uncultured_rumen_bacterium |
| OTU1103 | 4 | 67 | M4 | 0.485 | 0.000 | Peripheral nodes | Bacteria | Bacteroidetes | Bacteroidia | Bacteroidales | Marinilabiliaceae | g__uncultured | s__uncultured_rumen_bacterium |
| OTU2923 | 2 | 0 | M4 | -0.485 | 0.000 | Peripheral nodes | Bacteria | Bacteroidetes | Bacteroidia | Bacteroidales | Muribaculaceae | g__Muribaculaceae_ge | s__ |
| OTU2669 | 3 | 67 | M4 | 0.000 | 0.000 | Peripheral nodes | Bacteria | Bacteroidetes | Bacteroidia | Bacteroidales | Prevotellaceae | g__Prevotella_1 | s__uncultured_rumen_bacterium |
| OTU56 | 7 | 1216 | M4 | 1.940 | 0.000 | Peripheral nodes | Bacteria | Bacteroidetes | Bacteroidia | Bacteroidales | Rikenellaceae | g__Rikenellaceae_RC9_gut_group | s__ |
| OTU749 | 3 | 0 | M4 | 0.000 | 0.000 | Peripheral nodes | Bacteria | Bacteroidetes | Bacteroidia | Bacteroidales | Rikenellaceae | g__Rikenellaceae_RC9_gut_group | s__ |
| OTU584 | 5 | 260 | M4 | 0.970 | 0.444 | Peripheral nodes | Bacteria | Bacteroidetes | Bacteroidia | Bacteroidales | Rikenellaceae | g__Rikenellaceae_RC9_gut_group | s__uncultured_bacterium |
| OTU2590 | 1 | 0 | M4 | -0.970 | 0.000 | Peripheral nodes | Bacteria | Bacteroidetes | Bacteroidia | Bacteroidales | Rikenellaceae | g__Rikenellaceae_RC9_gut_group | s__uncultured_rumen_bacterium |
| OTU215 | 1 | 0 | M4 | -0.970 | 0.000 | Peripheral nodes | Bacteria | Firmicutes | Clostridia | Clostridiales | Ruminococcaceae | g__Saccharofermentans | s__uncultured_rumen_bacterium |
| OTU1679 | 1 | 0 | M4 | -0.970 | 0.000 | Peripheral nodes | Bacteria | Firmicutes | Erysipelotrichia | Erysipelotrichales | Erysipelotrichaceae | g__Erysipelotrichaceae_UCG-004 | s__uncultured_rumen_bacterium |
| OTU2526 | 3 | 133 | M5 | 0.777 | 0.000 | Peripheral nodes | Bacteria | Bacteroidetes | Bacteroidia | Bacteroidales | Muribaculaceae | g__Muribaculaceae_ge | s__uncultured_rumen_bacterium |
| OTU2753 | 2 | 256 | M5 | -0.111 | 0.000 | Peripheral nodes | Bacteria | Bacteroidetes | Bacteroidia | Bacteroidales | PeH15 | g__PeH15_ge | s__uncultured_rumen_bacterium |
| OTU2083 | 1 | 0 | M5 | -0.999 | 0.571 | Peripheral nodes | Bacteria | Bacteroidetes | Bacteroidia | Bacteroidales | Prevotellaceae | g__Prevotella_1 | s__uncultured_bacterium |
| OTU2418 | 2 | 195 | M5 | -0.111 | 0.000 | Peripheral nodes | Bacteria | Bacteroidetes | Bacteroidia | Bacteroidales | Prevotellaceae | g__Prevotellaceae_UCG-003 | s__uncultured_rumen_bacterium |
| OTU254 | 1 | 0 | M5 | -0.999 | 0.444 | Peripheral nodes | Bacteria | Bacteroidetes | Bacteroidia | Bacteroidales | Rikenellaceae | g__Rikenellaceae_RC9_gut_group | s__uncultured_rumen_bacterium |
| OTU1599 | 1 | 0 | M5 | -0.999 | 0.320 | Peripheral nodes | Bacteria | Bacteroidetes | Bacteroidia | Bacteroidales | Rikenellaceae | g__Rikenellaceae_RC9_gut_group | s__uncultured_rumen_bacterium |
| OTU2018 | 4 | 1063 | M5 | 1.665 | 0.000 | Peripheral nodes | Bacteria | Firmicutes | Clostridia | Clostridiales | Lachnospiraceae | g__Lachnospiraceae_ge | s__uncultured_rumen_bacterium |
| OTU472 | 3 | 377 | M5 | 0.777 | 0.000 | Peripheral nodes | Bacteria | Firmicutes | Clostridia | Clostridiales | Ruminococcaceae | g__Ruminococcus_2 | s__uncultured_bacterium |
| OTU178 | 3 | 5 | M6 | 0.642 | 0.000 | Peripheral nodes | Bacteria | Bacteroidetes | Bacteroidia | Bacteroidales | Bacteroidales BS11 gut group | g__Bacteroidales_BS11_gut_group_ge | s__uncultured_rumen_bacterium |
| OTU2581 | 1 | 0 | M6 | -1.155 | 0.000 | Peripheral nodes | Bacteria | Bacteroidetes | Bacteroidia | Bacteroidales | Prevotellaceae | g__Prevotellaceae_UCG-003 | s__uncultured_rumen_bacterium |
| OTU1795 | 4 | 9 | M6 | 1.541 | 0.000 | Peripheral nodes | Bacteria | Bacteroidetes | Bacteroidia | Bacteroidales | Rikenellaceae | g__Rikenellaceae_RC9_gut_group | s__uncultured_bacterium |
| OTU2523 | 2 | 0 | M6 | -0.257 | 0.500 | Peripheral nodes | Bacteria | Bacteroidetes | Bacteroidia | Bacteroidales | Rikenellaceae | g__Rikenellaceae_RC9_gut_group | s__uncultured_bacterium |
| OTU520 | 3 | 9 | M6 | 0.642 | 0.500 | Peripheral nodes | Bacteria | Bacteroidetes | Bacteroidia | Bacteroidales | Rikenellaceae | g__Rikenellaceae_RC9_gut_group | s__uncultured_rumen_bacterium |
| OTU1059 | 1 | 0 | M6 | -1.155 | 0.000 | Peripheral nodes | Bacteria | Bacteroidetes | Bacteroidia | Bacteroidales | Rikenellaceae | g__Rikenellaceae_RC9_gut_group | s__uncultured_rumen_bacterium |
| OTU2654 | 2 | 0 | M6 | -0.257 | 0.000 | Peripheral nodes | Bacteria | Firmicutes | Clostridia | Clostridiales | Ruminococcaceae | g__Ruminococcaceae_UCG-005 | s__uncultured_rumen_bacterium |
| OTU2327 | 3 | 67 | M7 | 0.000 | 0.000 | Peripheral nodes | Bacteria | Bacteroidetes | Bacteroidia | Bacteroidales | Bacteroidales RF16 group | g__Bacteroidales_RF16_group_ge | s__uncultured_bacterium |
| OTU1434 | 2 | 195 | M7 | -0.535 | 0.000 | Peripheral nodes | Bacteria | Bacteroidetes | Bacteroidia | Bacteroidales | Prevotellaceae | g__Prevotella_1 | s__uncultured_bacterium |
| OTU1318 | 3 | 132 | M7 | 0.000 | 0.480 | Peripheral nodes | Bacteria | Firmicutes | Clostridia | Clostridiales | Christensenellaceae | g__Christensenellaceae_R-7_group | s__ |
| OTU2058 | 6 | 533 | M7 | 1.604 | 0.000 | Peripheral nodes | Bacteria | Firmicutes | Clostridia | Clostridiales | Ruminococcaceae | g__Ruminococcaceae_UCG-014 | s__gut_metagenome |
| OTU842 | 1 | 0 | M7 | -1.069 | 0.245 | Peripheral nodes | Bacteria | Firmicutes | Clostridia | Clostridiales | Ruminococcaceae | g__Ruminococcus_2 | s__uncultured_rumen_bacterium |
| OTU3022 | 2 | 128 | M8 | -0.153 | 0.611 | Peripheral nodes | Bacteria | Bacteroidetes | Bacteroidia | Bacteroidales | Rikenellaceae | g__Rikenellaceae_RC9_gut_group | s__uncultured_rumen_bacterium |
| OTU769 | 3 | 67 | M8 | 0.614 | 0.000 | Peripheral nodes | Bacteria | Firmicutes | Clostridia | Clostridiales | Ruminococcaceae | g__ | s__ |
| OTU2008 | 1 | 0 | M8 | -0.920 | 0.480 | Peripheral nodes | Bacteria | Firmicutes | Clostridia | Clostridiales | Ruminococcaceae | g__Ruminococcaceae_ge | s__uncultured_rumen_bacterium |
| OTU1435 | 1 | 0 | M8 | -0.920 | 0.000 | Peripheral nodes | Bacteria | Firmicutes | Clostridia | Clostridiales | Ruminococcaceae | g__Ruminococcaceae_NK4A214_group | s__ |
| OTU95 | 4 | 74 | M8 | 1.381 | 0.444 | Peripheral nodes | Bacteria | Firmicutes | Clostridia | Clostridiales | Ruminococcaceae | g__Ruminococcaceae_NK4A214_group | s__uncultured_rumen_bacterium |
| OTU2824 | 1 | 0 | M9 | -1.000 | 0.320 | Peripheral nodes | Bacteria | Bacteroidetes | Bacteroidia | Bacteroidales | Prevotellaceae | g__Prevotella_1 | s__ |
| OTU1065 | 3 | 3 | M9 | 1.000 | 0.000 | Peripheral nodes | Bacteria | Firmicutes | Clostridia | Clostridiales | Christensenellaceae | g__Christensenellaceae_R-7_group | s__ |
| OTU596 | 1 | 0 | M9 | -1.000 | 0.000 | Peripheral nodes | Bacteria | Firmicutes | Clostridia | Clostridiales | Christensenellaceae | g__Christensenellaceae_R-7_group | s__uncultured_rumen_bacterium |
| OTU2067 | 3 | 3 | M9 | 1.000 | 0.000 | Peripheral nodes | Bacteria | Firmicutes | Clostridia | Clostridiales | Christensenellaceae | g__Christensenellaceae_R-7_group | s__uncultured_rumen_bacterium |
| OTU74 | 2 | 0 | M9 | 0.000 | 0.500 | Peripheral nodes | Bacteria | Firmicutes | Clostridia | Clostridiales | Lachnospiraceae | g__Lachnospiraceae_ge | s__Lachnospiraceae bacterium RM66 |
| OTU1906 | 2 | 0 | M10 | NA | NA | NA | Bacteria | Bacteroidetes | Bacteroidia | Bacteroidales | Prevotellaceae | g__Prevotella_1 | s__ |
| OTU157 | 2 | 0 | M10 | NA | NA | NA | Bacteria | Bacteroidetes | Bacteroidia | Bacteroidales | Prevotellaceae | g__Prevotella_1 | s__uncultured_rumen_bacterium |
| OTU2767 | 2 | 1 | M10 | NA | NA | NA | Bacteria | Bacteroidetes | Bacteroidia | Bacteroidales | Prevotellaceae | g__Prevotella_1 | s__uncultured_rumen_bacterium |
| OTU128 | 2 | 0 | M11 | -0.577 | 0.000 | Peripheral nodes | Bacteria | Bacteroidetes | Bacteroidia | Bacteroidales | Muribaculaceae | g__Muribaculaceae_ge | s__uncultured_bacterium |
| OTU65 | 3 | 73 | M11 | 1.155 | 0.000 | Peripheral nodes | Bacteria | Bacteroidetes | Bacteroidia | Bacteroidales | Rikenellaceae | g__Rikenellaceae_RC9_gut_group | s__Bacteroidales bacterium RM71 |
| OTU2684 | 2 | 0 | M11 | -0.577 | 0.000 | Peripheral nodes | Bacteria | Bacteroidetes | Bacteroidia | Bacteroidales | Rikenellaceae | g__Rikenellaceae_RC9_gut_group | s__uncultured_rumen_bacterium |
| OTU2955 | 1 | 0 | M12 | NA | NA | NA | Bacteria | Bacteroidetes | Bacteroidia | Bacteroidales | Prevotellaceae | g__Prevotellaceae_UCG-003 | s__uncultured_rumen_bacterium |
| OTU2088 | 1 | 0 | M12 | NA | NA | NA | Bacteria | Firmicutes | Erysipelotrichia | Erysipelotrichales | Erysipelotrichaceae | g__Erysipelotrichaceae_UCG-004 | s__uncultured_rumen_bacterium |
| OTU23 | 1 | 0 | M13 | NA | NA | NA | Bacteria | Firmicutes | Clostridia | Clostridiales | Ruminococcaceae | g__Ruminococcaceae_UCG-014 | s__uncultured_bacterium |
| OTU2219 | 1 | 0 | M13 | NA | NA | NA | Bacteria | Firmicutes | Clostridia | Clostridiales | Ruminococcaceae | g__uncultured | s__uncultured_rumen_bacterium |
| OTU1204 | 1 | 0 | M14 | NA | NA | NA | Bacteria | Bacteroidetes | Bacteroidia | Bacteroidales | Prevotellaceae | g__Prevotella_1 | s__ |
| OTU2333 | 1 | 0 | M14 | NA | NA | NA | Bacteria | Bacteroidetes | Bacteroidia | Bacteroidales | Prevotellaceae | g__Prevotella_1 | s__uncultured_rumen_bacterium |
| OTU2427 | 1 | 0 | M15 | NA | NA | NA | Bacteria | Firmicutes | Clostridia | Clostridiales | Lachnospiraceae | g__Lachnospiraceae_ND3007_group | s__uncultured_rumen_bacterium |
| OTU1412 | 1 | 0 | M15 | NA | NA | NA | Bacteria | Firmicutes | Clostridia | Clostridiales | Lachnospiraceae | g__Lachnospiraceae_XPB1014_group | s__uncultured_rumen_bacterium |
| OTU329 | 1 | 0 | M16 | NA | NA | NA | Bacteria | Firmicutes | Clostridia | Clostridiales | Lachnospiraceae | g__Lachnospiraceae_XPB1014_group | s__uncultured_bacterium |
| OTU2959 | 1 | 0 | M16 | NA | NA | NA | Bacteria | Firmicutes | Clostridia | Clostridiales | Lachnospiraceae | g__uncultured | s__ |
| OTU1424 | 1 | 0 | M17 | NA | NA | NA | Bacteria | Firmicutes | Clostridia | Clostridiales | Lachnospiraceae | g__Lachnospiraceae_ND3007_group | s__uncultured Lachnospiraceae bacterium |
| OTU1922 | 1 | 0 | M17 | NA | NA | NA | Bacteria | Firmicutes | Clostridia | Clostridiales | Lachnospiraceae | g__Lachnospiraceae_ND3007_group | s__uncultured_rumen_bacterium |
| OTU796 | 2 | 67 | M18 | 0.707 | 0.000 | Peripheral nodes | Bacteria | Firmicutes | Clostridia | Clostridiales | Lachnospiraceae | g__Lachnospiraceae_AC2044_group | s__uncultured_bacterium |
| OTU2096 | 1 | 0 | M18 | -0.707 | 0.000 | Peripheral nodes | Bacteria | Firmicutes | Clostridia | Clostridiales | Lachnospiraceae | g__Lachnospiraceae_FCS020_group | s__uncultured_rumen_bacterium |
| OTU1896 | 1 | 0 | M19 | NA | NA | NA | Bacteria | Bacteroidetes | Bacteroidia | Bacteroidales | Prevotellaceae | g__Prevotellaceae_UCG-003 | s__uncultured_rumen_bacterium |
| OTU2432 | 1 | 0 | M19 | NA | NA | NA | Bacteria | Firmicutes | Clostridia | Clostridiales | Ruminococcaceae | g__Saccharofermentans | s__ |

Table S5. The cumulative relative abundance of the modules

| Module | Breed | Dietary energy level (MJ/kg DM) | | | | SEM | *P*-value^*^ | | | |
| --- | --- | --- | --- | --- | --- | --- | --- | --- | --- | --- |
|  |  | 6.73 | 7.65 | 8.57 | 9.49 |  | Breed | E-L | E-Q | E-C |
| M1 | H | 12.71 | 12.85 | 8.82 | 9.61 | 3.476 | 0.364 | 0.264 | 0.959 | 0.787 |
|  | T | 9.19 | 8.11 | 8.30 | 6.11 |  |  | 0.825 ^x^ | 0.839 ^x^ | 0.516 ^x^ |
| M2 | H | 4.81 | 8.05 | 4.81 | 5.51 | 1.304 | 0.148 | 0.408 | 0.141 | 0.021 |
|  | T | 3.90 | 6.99 | 3.25 | 3.18 |  |  | 0.567 ^x^ | 0.862 ^x^ | 0.991 ^x^ |
| M3 | H | 8.77 | 3.24 | 8.47 | 3.39 | 3.089 | 0.194 | 0.592 | 0.932 | 0.326 |
|  | T | 2.90 | 2.99 | 2.64 | 3.05 |  |  | 0.576 ^x^ | 0.989 ^x^ | 0.263 ^x^ |
| M4 | H | 1.39 | 2.43 | 5.35 | 2.29 | 1.259 | 0.098 | 0.106 | 0.160 | 0.375 |
|  | T | 3.43 | 4.77 | 5.21 | 5.69 |  |  | 0.837 ^x^ | 0.343 ^x^ | 0.250 ^x^ |
| M5 | H | 0.77 | 1.29 | 0.65 | 1.78 | 0.800 | 0.120 | 0.152 | 0.331 | 0.981 |
|  | T | 1.96 | 1.08 | 2.44 | 3.19 |  |  | 0.593 ^x^ | 0.646 ^x^ | 0.254 ^x^ |
| M6 | H | 1.16 | 0.62 | 1.08 | 0.55 | 1.552 | 0.009 | 0.126 | 0.634 | 0.662 |
|  | T | 6.97 | 5.73 | 2.36 | 3.26 |  |  | 0.191 ^x^ | 0.631 ^x^ | 0.398 ^x^ |
| M7 | H | 0.37 | 1.46 | 1.66 | 6.01 | 1.376 | 0.074 | 0.049 | 0.513 | 0.526 |
|  | T | 0.00 | 0.58 | 0.52 | 0.46 |  |  | 0.082 ^x^ | 0.322 ^x^ | 0.618 ^x^ |
| M8 | H | 2.72 | 4.40 | 5.30 | 5.35 | 1.278 | 0.014 | 0.022 | 0.666 | 0.733 |
|  | T | 5.66 | 6.25 | 8.22 | 8.84 |  |  | 0.737 ^x^ | 0.650 ^x^ | 0.745 ^x^ |
| M9 | H | 0.86 | 1.29 | 0.59 | 6.37 | 1.841 | 0.807 | 0.199 | 0.230 | 0.452 |
|  | T | 2.20 | 1.88 | 1.43 | 2.28 |  |  | 0.179 ^x^ | 0.427 ^x^ | 0.598 ^x^ |
| M10 | H | 0.53 | 2.18 | 0.57 | 1.00 | 1.180 | 0.115 | 0.694 | 0.579 | 0.162 |
|  | T | 2.65 | 3.76 | 1.89 | 2.37 |  |  | 0.731 ^x^ | 0.854 ^x^ | 0.994 ^x^ |
| M11 | H | 0.40 | 0.53 | 0.26 | 0.26 | 0.202 | 0.069 | 0.548 | 0.192 | 0.884 |
|  | T | 0.57 | 0.78 | 0.92 | 0.50 |  |  | 0.600 ^x^ | 0.370 ^x^ | 0.351 ^x^ |

^*^E-L = linear effect of dietary energy; E-Q = quadratic effect of dietary energy; E-C = cubic effect of dietary energy.

^x^*P*-value for the interaction of dietary energy effect with the breed.

H, small-tailed Han sheep; T, Tibetan sheep.


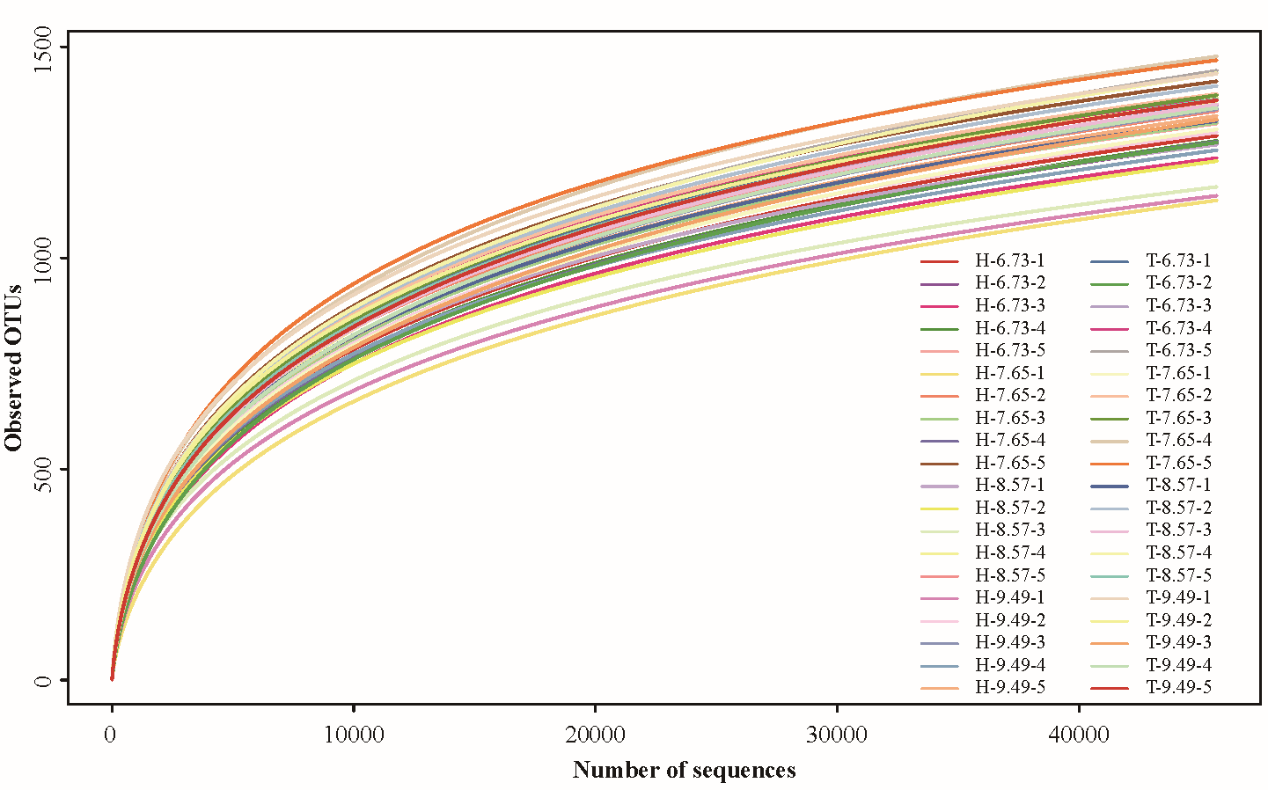


Figure S1. Rarefaction curves of OTUs clustered at 97% similarity level across different samples


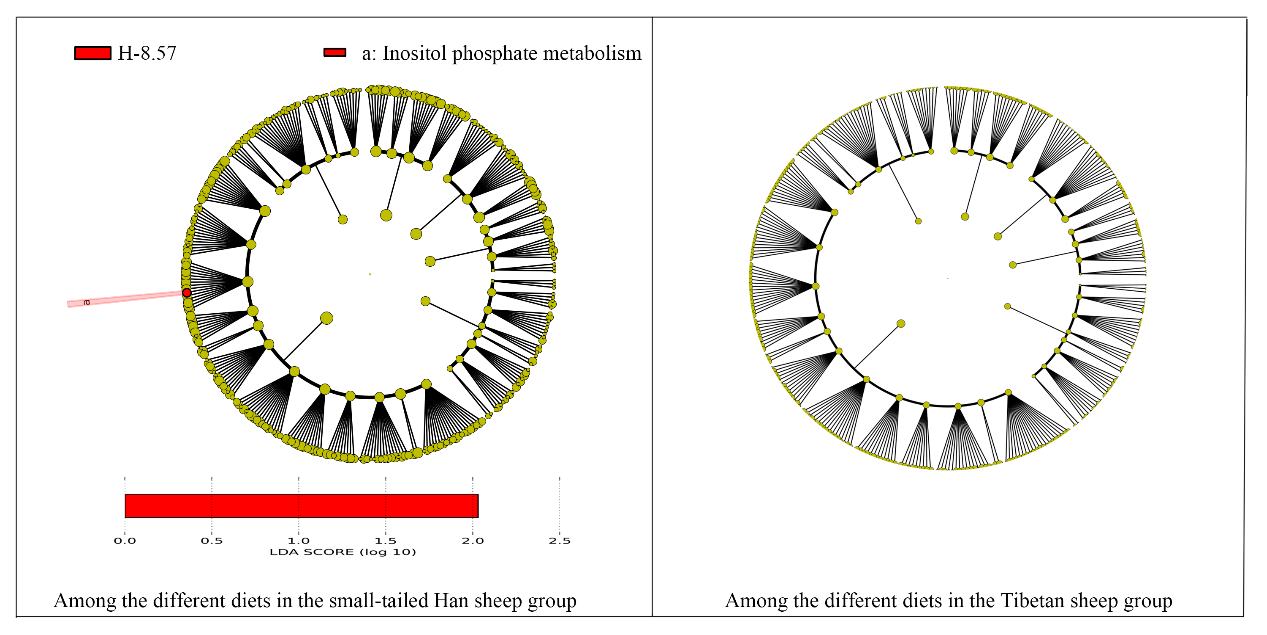


Figure S2. LEfSe analysis was conducted on the cladogram and LDA plots of metagenomic functional predictions of KEGG Pathway among the different dietary treatments in the Small-tailed Han sheep and Tibetan sheep breeds.
